# The Therapeutic Benefits of Nanoencapsulation in Drug Delivery to the Anterior Segment of the Eye: A Systematic Review

**DOI:** 10.3389/fphar.2022.903519

**Published:** 2022-05-13

**Authors:** Madhavi Bhandari, Sanko Nguyen, Mazyar Yazdani, Tor Paaske Utheim, Ellen Hagesaether

**Affiliations:** ^1^ Department of Life Sciences and Health, Faculty of Health Sciences, Oslo Metropolitan University, Oslo, Norway; ^2^ Department of Medical Biochemistry, Oslo University Hospital, Oslo, Norway; ^3^ Department of Ophthalmology, Oslo University Hospital, Oslo, Norway

**Keywords:** ocular drug delivery, topical administration, nanoparticle, drug delivery, encapsulation, animal studies, *in vivo* efficacy

## Abstract

**Background:** Although numerous nanoparticle formulations have been developed for ocular administration, concerns are being raised about a possible mismatch between potential promises made by the field of nanoparticle research and demonstration of actual therapeutic benefit. Therefore, the primary focus of this present review was to critically assess to what extent nanoencapsulation of ocular drugs improved the therapeutic outcome when treating conditions in the anterior segment of the eye.

**Methods:** A systematic search was conducted using Medline, PubMed, and Embase databases as well as Google Scholar for published peer-reviewed articles in English focusing on conventional nanoparticles used as drug delivery systems to the anterior segment of the eye in *in vivo* studies. The major therapeutic outcomes were intraocular pressure, tear secretion, number of polymorphonuclear leucocytes and pupil size. The outcome after encapsulation was compared to the non-encapsulated drug.

**Results:** From the search, 250 results were retrieved. Thirty-eight studies met the inclusion criteria. Rabbits were used as study subjects in all but one study, and the number of animals ranged from 3 to 10. Coated and uncoated liposomes, lipid-based and polymeric nanoparticles, as well as micelles, were studied, varying in both particle size and surface charge, and encapsulating a total of 24 different drugs, including 6 salts. The majority of the *in vivo* studies demonstrated some improvement after nanoencapsulation, but the duration of the benefit varied from less than 1 h to more than 20 h. The most common *in vitro* methods performed in the studies were drug release, transcorneal permeation, and mucin interaction.

**Discussion:** Nanoparticles that are small and mucoadhesive, often due to positive surface charge, appeared beneficial. Although *in vitro* assays can unravel more of the hidden and sophisticated interplay between the encapsulated drug and the nanoparticle structure, they suffered from a lack of *in vitro*—*in vivo* correlation. Therefore, more research should be focused towards developing predictive *in vitro* models, allowing rational design and systematic optimization of ocular nanoparticles with minimal animal experimentation.

## 1 Introduction

The burden of eye conditions is expected to increase in coming decades with a growing population and aging, behavioral and lifestyle changes (WHO, 2019). While some eye conditions, such as glaucoma, can cause vision impairment and blindness, many, such as dry eye disease (DED), ocular infection and inflammation, usually do not. Yet, these conditions are among the leading reasons for seeking medical care causing significant financial strain on the health care system (WHO, 2019) and negatively affecting quality of life (Dhouib et al., 2021; Morthen et al., 2022). Currently, there is a vast array of ocular therapeutics available, however, the main challenge has been to develop a delivery method that can overcome the anatomical and physiological barriers of the eye to improve their efficacy. Therefore, there is a growing demand for the development of safe and effective ocular drug delivery systems in the treatment of eye conditions.

The eye is a delicate organ comprised of the anterior and posterior segments (Dave et al., 2021). Conditions affecting the anterior segment of the eye such as glaucoma (Arroyo et al., 2018), DED (Chen et al., 2021), and inflammation (Katara et al., 2019) can be treated using ocular implants, intracameral or subconjunctival injections, topical and oral administration of ocular drugs. However, many of these methods are either invasive or can cause serious systemic side effects (Dave et al., 2021). Topical application remains the easiest, least invasive and safest route to deliver therapeutics (Jumelle et al., 2020; Dave et al., 2021) despite low bioavailability (Jumelle et al., 2020).

The main barrier to ocular drug delivery is the tear film. Based on the classical model, this transparent fluid is comprised of three-layers (∼10 µM thick). The outermost oily layer contains lipids; the intermediate aqueous layer contains salts, mucins, proteins, and enzymes; and the innermost mucus layer contains lysozymes and glycocalyx. Immediately upon application, ocular drugs are diluted by the tear film produced mainly by the lacrimal and Meibomian glands as a protection mechanism of the eye (Nichols et al., 1985; Yañez-Soto et al., 2014). Additionally, tear film turnover (normally ∼1–3 μl/min) that increases upon topical application can cause rapid clearance of drug molecules (within 1–2 min) via nasolacrimal drainage (Worakul and Robinson, 1997) leading to low ocular retention and absorption. The lipid and aqueous layers in the tear film impede permeation of hydrophilic and hydrophobic drugs, respectively. Lysozymes present in the tear film can also degrade the administered drugs (Worakul and Robinson, 1997; Campos et al., 2020). Another barrier is the limited precorneal surface area. It is estimated that only about 30 µl of eye drops can be applied onto the ocular surface, most of which is instantly eliminated during the first reflex blinking (Ghate and Edelhauser, 2006). Besides, tight epithelial junctions in cornea, conjunctival and scleral tissues represent a major physical barrier for drug diffusion through anterior ocular tissues, a prerequisite for pharmacological treatment of glaucoma and uveitis, for instance (Dastjerdi et al., 2011). Moreover, efflux pumps (Karla et al., 2009) and cytochrome P450 (Zhang et al., 2008) present in the corneal epithelium negatively influence drug delivery. All these barriers cause low bioavailability in which less than 5% of the drugs administered via the topical route reach the target tissues (Jumelle et al., 2020). Consequently, high drug concentrations and frequent administration are often required in traditional topical formulations to achieve the desired therapeutic effects. This is time consuming and predisposes patients to several adverse effects, such as temporary blurred vision, ocular discomfort, and damage to the ocular surface. Especially upon long-term use and in chronic eye conditions, these disadvantages lead to low patient compliance and treatment failure (Gholizadeh et al., 2021; Patel et al., 2022).

A variety of drug delivery technologies and systems, such as prodrugs, *in situ* gels, cul-de-sac inserts (Lacrisert^®^) (Abdelkader et al., 2021) and carrier systems using nanoparticles, have been investigated and developed over the past decades to address the shortcomings associated with conventional eye formulations (Jumelle et al., 2020; Gholizadeh et al., 2021). Among them, nanoparticle-based systems have gained significant attraction due to their potential to improve ocular retention (Janagam et al., 2017; Jumelle et al., 2020). In addition, encapsulation into nanoparticles may prevent biologic and enzymatic degradation of drugs and, as a result, lower concentrations may be sufficient to achieve the desired therapeutic effects (Formica et al., 2021; Patel et al., 2022). The surface properties of nanoparticles, such as hydrophobicity or hydrophilicity and charge, can be easily tuned to increase the affinity towards ocular tissues and enable closer contact with the mucin layer on the ocular surface (Janagam et al., 2017; Patel et al., 2022). Nanoparticles can also be modified to release the encapsulated drug in a sustained manner and enhance drug permeability (Jumelle et al., 2020) using excipients such as chitosan (Wadhwa et al., 2010). Owing to their small size (<1 µM), nanoparticulate formulations do not pose discomfort or irritation to the eye and they can be easily prepared as liquid dosage forms, such as eye drops, for ease of administration (Jumelle et al., 2020; Gholizadeh et al., 2021). Nanoparticle-based systems offer great potential when it comes to ocular drug delivery. However, less is known whether the recent nanomedicinal developments for ocular therapy do live up to their promises.

A few nanoparticle-based ophthalmic products are currently commercially available. These include: 1) Inveltys^®^, which comprises mucus-penetrating polymeric nanoparticles with loteprednol etabonate for treatment of inflammation and pain after ocular surgery; 2) the cationic nanoemulsion Cyclokat^®^; 3) the anionic nanoemulsion Restasis^®^; and 4) the nanomicellar formulation Cequa^®^, all containing cyclosporine A for DED treatment (Natesan et al., 2020). In the literature, a great variety of different lipid and polymeric nanoparticles, and combinations thereof, have been described and many are under development for drug delivery to the anterior segment of the eye. Most of these are based on materials that are biocompatible and generally recognized as safe both from natural sources, such as chitosan, hyaluronic acid, alginate, and gelatin, and from synthetic origin, such as polymethacrylate-based copolymers (Eudragit^®^), poly (lactic-co-glycolic acid) (PLGA), and polycaprolactone (Jumelle et al., 2020). Further, many clinical trials are underway for different eye diseases using mucus penetrating nanoparticles. These include: 1) KPI-121-C-001, which is under Phase III clinical trials for ocular infections, irritations, and inflammations; 2) KPI-121-C-002, which is under Phase II clinical evaluation for dry eye; 3) KPI-121-C-003, which is intended for blepharitis and is in Phase II trial; and 4) KPI-121-C-004, which is currently under investigation for retinal vein occlusion and diabetic macular edema (Campos et al., 2020).

**FIGURE 1:**
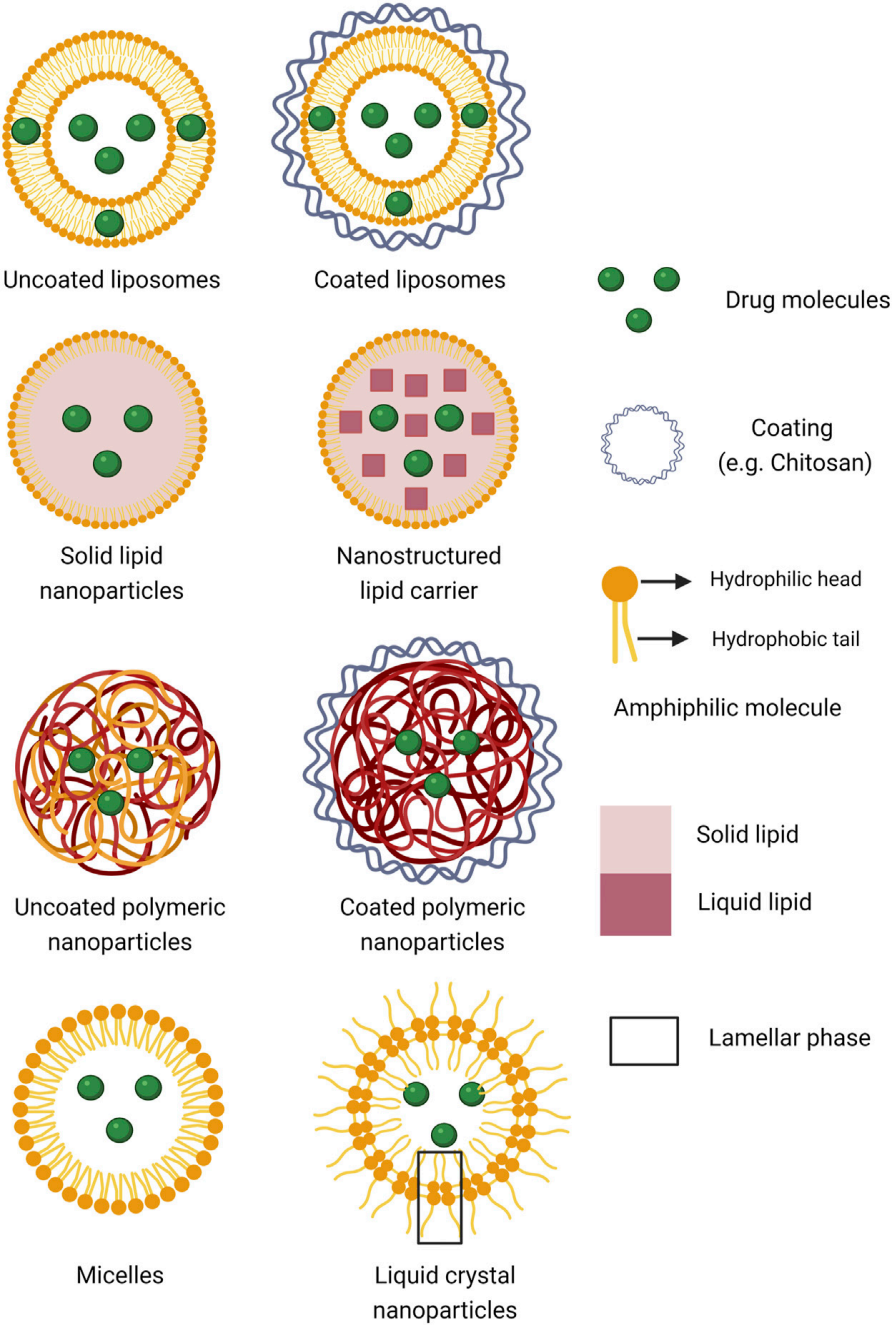
Schematic presentation of the different types of nanoparticulate systems for drug encapsulation scrutinized in the present analysis (Created with BioRender.com).

Despite the interest and excitement in nanomedicine, critical opinion letters have recently emerged in highly ranked pharmaceutical journals raising concerns about their lack of clinical translation, further, highlighting the importance of relevant and accurate data (Allen, 2019; Park, 2019). The main objective of this systematic review was, therefore, to scrutinize the *in vivo* therapeutic outcomes of “common” nanoparticles (Figure 1) for treatment of conditions in the anterior segment of the eye and present our critical analyses as to whether nanoparticular encapsulation improved ocular drug treatment when compared to non-encapsulated drug administration. If granted therapeutic improvement, we also wanted to identify the most successful types of nanoparticles and search for possible patterns of physicochemical parameters, e.g., surface charge of nanoparticles and nature of encapsulated drug, that may enhance their *in vivo* performance. A final objective was to study the correlation between *in vitro* and *in vivo* behavior of the nanoparticles.

## 2 Methods

A systematic search for published peer-reviewed articles focusing on drug delivery to the anterior segment of the eye was performed in Medline and Embase databases using the Ovid interface in October 2021. The search was limited to articles written in English, but not by publication date. The systematic search was thematically structured into four categories: 1) nanoparticles, 2) used as a drug delivery system, 3) installed on the ocular surface, 4) in *in vivo* systems. Both “free text words” and “medical subject headings (MeSH) terms”—used by the databases for indexing articles—were used for the search. Overlapping search words were used in each category, which were combined with “OR” to broaden the search within the category. The search results from all four categories were then combined with “AND” to narrow the search (Table 1). Hits of the type “original article” were screened based on inclusion and exclusion criteria (Table 2). Two authors reviewed each article independently, and a third one was involved in case of disagreement. Hits of the type “review article” were first screened for relevance, based on title and abstract, by two authors. Then the bibliographies of the most relevant review articles were scrutinized for relevant original articles, using the procedure already described for original articles. Four authors screened at least 10 bibliographies each, covering 48 bibliographies in total. Finally, a non-systematic search was conducted in Google Scholar to look for studies that were missing from the systematic search.

**TABLE 1:** Systematic search thematically structured into four categories of “MeSH terms” and “free text words” used for literature search in Medline and Embase databases using the Ovid interface. The “MeSH terms” and “free text words” in each category were combined with “OR” to broaden the search within the category. The search results from all four categories were then combined with “AND” to narrow the search.

AND				
**OR**	1	2	3	4
	Nanoparticle^*^.mp. or \ (Nanostructures\ Nanocapsules\)	Drug delivery systems \	(Anterior adj5 eye).mp.	*In vivo*.mp
	Nanocarrier^*^.mp.	Drug carriers \	(Ocular adj5 surface).mp.	Animal experimentation \
	Nanomedicine \	Drug adj3 delivery.mp	(Eye adj5 surface).mp.	Humans \
				(Cornea adj5 surface).mp.	Animals\ Mice\

mp - free text words; \ - MeSH, terms.

* - truncation; adj3/adj5—search for words standing close (3 and 5 specifies the maximum number of words separating the search terms in any order).

**TABLE 2:** Criteria for screening of original research articles.

Inclusion	Exclusion
1. Nanoparticles encapsulating a therapeutic agent (e.g., drug, lubricant, water, macromolecules) physically, but not *via* a covalent linkage	1. The outcome was limited to bioavailability or evaluation of pharmacokinetic parameters
	2. The therapeutic outcome was assessed qualitatively, e.g., *in vivo* anti-inflammatory efficacy was assessed by assigning an ocular inflammation score (Draize eye test)
2. Nanoparticles with/without surface receptors and installed on the ocular surface from where the drug must be released or aimed at treating a condition occurring on the ocular surface	3. Formulations describing microspheres, cyclodextrins, dendrimers, nanowafers, microemulsions, as well as formulations containing a multitude of excipients, such as nanoemulsions, where the possible effect of excipients cannot be separated from the effect of nanoencapsulation
	4. Formulations intended for DNA/RNA delivery and transfection studies, where nanoparticles transport the nucleic acids into the cells
3. Nanoparticles studied in *in vivo* systems where the “free therapeutic agent” (or simple preparation of the drug or standard commercially available product such as eye drops) is used as a control to monitor the therapeutic outcome	5. Unconventional or rarely used nanoparticle core, for which results from only one research group was available. Novel excipients not generally recognized as safe (GRAS listed)
	6. Articles published before the year 2002

Our research question, search strategy and inclusion/exclusion criteria can be framed to the PICO process as described in the following. The population (P) was human and animal subjects, both healthy and with conditions in the anterior segment of the eye. The intervention (I) was drug encapsulated in conventional nanoformulations installed on the ocular surface. The comparison (C) was the non-encapsulated (“free”) therapeutic agent. The outcome (O) was initially not limited to any specific clinical parameter, as we wanted the search to include a variety of ocular conditions. However, the outcome was limited to a quantifiable therapeutic outcome of a condition occurring on the ocular surface or after the drug has been released on the ocular surface.

Two authors retrieved and synthesized the results together. The therapeutic outcome of encapsulation within all reviewed ocular conditions was compared to the non-encapsulated drug including in some cases the commercial reference. In DED, ocular inflammation, and endotoxin-induced uveitis, this comparison was expressed as a ratio further used to rank the nanoparticles based on their performances. For DED, we focused on tear (secretion) volume and higher ratios were interpreted as improved therapeutic effect of nanoparticles. In ocular inflammation and endotoxin-induced uveitis, we focused on the number of polymorphonuclear cells, and lower ratios suggested better treatment outcome with the nanoparticles. An emphasis was given to the polymorphonuclear leucocytes values at specific time points that reflect a high degree of inflammation, as the effect of treatment should be greatest when the inflammation is at a maximum. For pupillary constriction/dilation evaluation, the ability of nanoparticles to extend the amount or duration of pupil diameter was considered following topical application to rabbit eyes. In case of glaucoma, the results were presented in various ways either as intraocular pressure (IOP) reduction (or its percentage) or IOP values. Due to such varied ways of data presentation in glaucoma studies, we, for ease of evaluation, ranked the therapeutic outcome within each data set by four categories of *in vivo* efficacy. These categories were based on the superior (1), good (2), moderate (3), and marginal (4) performance of the nanoparticles. The ranking criteria are summarized in Table 3.

**TABLE 3:** Categorization of nanoparticle efficacy based on response criteria when evaluating *in vivo* efficacy of nanoencapsulation of anti-glaucoma drugs when compared to non-encapsulated drug, in the reviewed studies.

Nanoparticle efficacy categories	Response criteria	Reference
Nanoparticles prolonged the IOP reduction by ≥ 15% for		
1. Superior	≥4 h	Arroyo et al. (2018) ^a^
		Li et al. (2013)
2. Good	∼2 h	Abd-Elsalam and ElKasabgy, (2019)
		Wang et al. (2014) ^a^
3. Moderate	<1 h	Arroyo et al. (2018) ^a^
		Wang et al. (2014) ^a^
4. Marginal	Less than 0 h	None
Nanoparticles prolonged the IOP reduction by ≥ 5 mmHg for		
1. Superior	>12 h	Bhagav et al. (2011)
		Leonardi et al. (2015) ^a^
		Wadhwa et al. (2010) ^a^
2. Good	∼4–6 h	Manchanda and Sahoo, (2018)
		Verma et al. (2013)
		Wadhwa et al. (2010) ^a^
		Youshia et al. (2012)
3. Moderate	3 h	None
4. Marginal	≤1 h	Leonardi et al. (2015) ^a^
		Musumeci et al. (2013)
Nanoparticles increased the IOP reduction by ≥ 20% for		
1. Superior	>20 h	Khan et al. (2018)
		Shokry et al. (2018) ^a^
2. Good	4–12 h	Warsi et al. (2014)
3. Moderate	3–4 h	Natesan et al. (2017)
		Ameeduzzafar et al. (2014)
4. Marginal	∼2 h	Rubenicia et al. (2021)
		Tan et al. (2017)
		Wang et al. (2018)
		Shokry et al. (2018) ^a^

a Some studies evaluated more than one type of nanoparticles for *in vivo* efficacy. When such studies were listed in more than one category, the explanation is briefly described below.

Conventional liposomes performed superior, and deformable liposomes moderately (Arroyo et al., 2018).

Chitosan-coated solid lipid nanoparticles performed well, and plain solid lipid nanoparticles moderately (Wang et al., 2014).

Solid lipid nanoparticles modified with palmitic acid performed superior, and unmodified solid lipid nanoparticles performed marginal (Leonardi et al., 2015).

Hyaluronic acid modified chitosan nanoparticles performed superior, and unmodified nanoparticles performed well (Wadhwa et al., 2010).

Nanoparticles crosslinked for 16 h performed superior, and nanoparticles crosslinked for 8 h performed marginal (Shokry et al., 2018).

For reporting the studies, we consulted and followed the PRISMA guidelines where relevant. PRISMA checklist primarily focuses on the reporting of studies assessing the effects of intervention, often in a clinical setting aimed at a particular condition. The studies we found evaluated a technological intervention applied in several different conditions and studied using a small number of animal subjects.

## 3 Results

**FIGURE 2:**
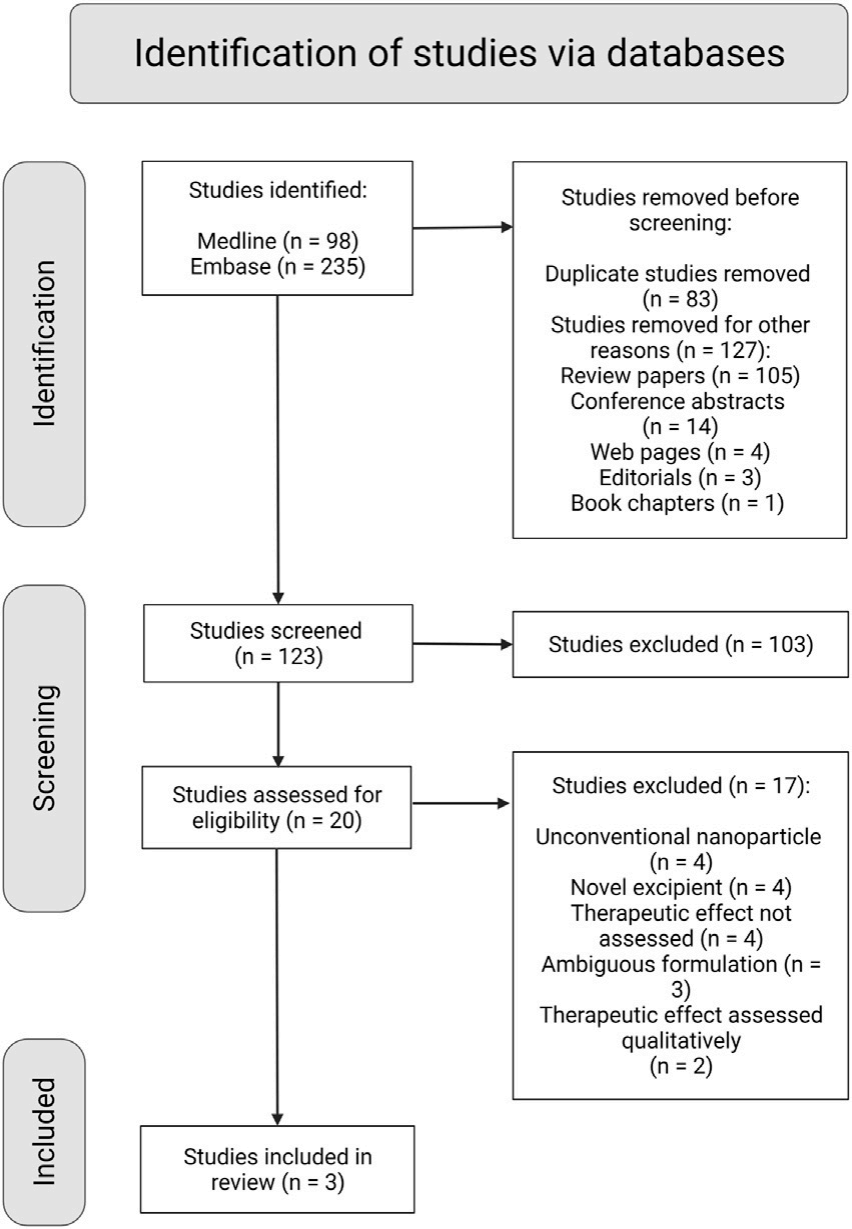
PRISMA flow diagram with schematic representation of the screening and selection process of studies retrieved from the systematic search (Created with BioRender.com).

We retrieved 250 hits from our systematic search in Medline and Embase databases: 123 original research articles and 105 review articles, of which 48 reviews were regarded relevant. From the 123 original research articles, only three met all inclusion criteria as shown in the flowchart in Figure 2. After screening the bibliography of the 48 review articles, 35 more original research articles meeting all inclusion criteria were identified. Finally, 38 articles in total met all the inclusion criteria (Table 4). The search from Google Scholar did not yield additional articles.

**TABLE 4:** Summary of *in vivo* studies on nanoparticle-based systems used for drug delivery to anterior segment of the eye.

Type of Nanoparticle; drug	Principle of preparation; main excipients	Characterization studies; size, ζ-potential	Animal model (*n* = number of animals) and key *in vivo* results	Reference
Glaucoma				
Liposomes; timolol maleate	Lipid film-hydration	Drug release, transcorneal permeation	Normotensive rabbit (*n* = 10 rabbits/20 eyes) Maximal IOP reduction: ∼12% observed after 4 h with commercial product. AUC_0_^7h^ (%h) value: ∼32	Arroyo et al. (2018)
	Conventional: phosphatidylcholine, cholesterol	151 nm, −3 mV	Maximal IOP reduction: ∼23% observed after 7 h AUC_0_^7h^ (%h) value: ∼86	
	Deformable: additional deoxycholate, ethanol	355 nm, −3 mV	Maximal IOP reduction: ∼20% observed after 5 h AUC_0_^7h^ (%h) value: ∼64	
Liquid crystal nanoparticles; pilocarpine nitrate	Single emulsion followed by solvent evaporation; glyceryl monoolein, poloxamer 407	Drug release, transcorneal permeation 202 nm	Normotensive rabbit (*n* = 6) Maximal IOP reduction: ∼42% observed after 2 h with commercial product. An effect was observed for 8 h. Maximal IOP reduction: ∼59% observed after 5 h with nanoparticles. An effect was observed for up to 12 h	Li et al. (2013)
Liposomes coated with chitosan; agomelatine	Lipid film-hydration	Drug release, mucin interaction	Hypertensive rabbit (*n* = 8) Maximal IOP reduction: 50% observed after 1 h with drug solution. AUC_0-8_: 108 mmHg.h	Abd-Elsalam and ElKasabgy, (2019)
	oleic acid, sorbitan monooleate, chitosan	1391 nm, +35 mV	Maximal IOP reduction: 55% observed after 2 h. AUC_0-8_: 213 mmHg.h	
	additional oleylamine	790 nm, +31 mV	Maximal IOP reduction: 73% after 2 h. AUC_0-8_: 301 mmHg h	
Solid lipid nanoparticles; methazolamide	Single emulsion followed by solvent evaporation	Drug release, transcorneal permeation	Normotensive rabbit (*n* = 6) Maximal IOP reduction: 38% with commercial product. An effect was observed for ∼6 h. AUC_0-8_: 171 mmHg h	Wang et al. (2014)
	glyceryl monostearate, lecithin, PEG400	199 nm, −21 mV	Maximal IOP reduction: 28%. An effect was observed for ∼6 h. AUC_0-8_: 127 mmHg h	
	additional chitosan coating	253 nm, +31 mV	Maximal IOP reduction: 43%. An effect was observed for >8 h. AUC_0-8_: 246 mmHg h	
Polymeric nanoparticles; brimonidine tartrate	Double emulsion followed by solvent evaporation	Drug release	Hypertensive rabbit (*n* = 3) Maximal IOP reduction: 9 mmHg observed after 1 h with commercial product. An effect was observed for 6 h. AUC_ΔIOPvs.t_: 38 mmHg h	Bhagav et al. (2011)
	Eudragit^®^ and poloxamer (lower ratio), lecithin	220 nm	Maximal IOP reduction: 8 mmHg observed after 3 h. An effect was observed for 36 h. AUC_ΔIOP vs. t_: 136 mmHg h	
	Eudragit^®^ and poloxamer (higher ratio), lecithin	325 nm	Maximal IOP reduction: 8 mmHg observed after 3 h. An effect was observed for 72 h. AUC_ΔIOP vs. t_: 268 mmHg h	
Solid lipid nanoparticles; melatonin	Quasi-emulsion followed by solvent evaporation	Drug release	Normotensive rabbit (*n* = 4) Maximal IOP reduction: 3–4 mmHg observed after 2 h with drug solution. The effect diminished within the next 2 h	Leonardi et al. (2015)
	Softisan^®^100, dodecyldimethylammonium bromide	182 nm, +59 mV	Maximal IOP reduction: <2.5 mmHg throughout the experiment	
	additional stearic acid	237 nm, +58 mV	Maximal IOP reduction: ∼7 mmHg observed after 1 h, with a slight increase within the next 5 h	
	additional palmitic acid	223 nm, +60 mV	Maximal IOP reduction: ∼7 mmHg observed after 6–8 h, and a reduction of >5 mmHg was sustained for the next 16 h	
Polymeric nanoparticles; timolol maleate and dorzolamide hydrochloride	Ionotropic gelation	Drug release, trans-corneal permeation, mucin interaction	Normotensive rabbit (*n* = 6). Maximal IOP reduction: 6 mmHg after 3–4 h with commercial product, gradual decrease for the next 68 h	Wadhwa et al. (2010)
	chitosan, tripolyphosphate	118 nm, +29 mV	Maximal IOP reduction: 9 mmHg observed after 4–8 h, followed by a gradual decrease for the next 64 h	
	additional hyaluronic acid	320 nm, +33 mV	Maximal IOP reduction: 10 mmHg observed after 4–12 h, followed by a gradual decrease for the next 60 h	
Polymeric nanoparticles; dorzolamide hydrochloride	Ionotropic gelation	Drug release, trans-corneal permeation, mucin interaction	Normotensive rabbit (*n* = 3) Maximal IOP reduction: 7–8 mmHg observed after 1 h with drug solution, followed by a gradual decrease for 3 h	Manchanda and Sahoo, (2018)
	chitosan, dextran sulphate	183 nm, +43 mV	Maximal IOP reduction: 13–14 mmHg observed after 3 h, followed by a slight decrease within the next 2 h	
	chitosan, tripolyphosphate	172 nm, +37 mV	Maximal IOP reduction: 12–13 mmHg observed after 3–4 h, followed by a gradual decrease within the next hour	
Polymeric nanoparticles; acetazolamide	Desolvation followed by evaporation; Eudragit^®^	Drug release 92–98 nm, + (16–19) mV	Normotensive rabbit (*n* = 6) Maximal IOP reduction: ∼3 mmHg with drug solution. An effect was observed for ∼2.5 h. Maximal IOP reduction: ∼5 mmHg with nanoparticles. An effect was observed for ∼8 h	Verma et al. (2013)
Nanostructured lipid matrix; methazolamide	Single emulsion followed by solvent evaporation; Compritol^®^, cetostearyl alcohol, stearylamine and	Drug release	Normotensive rabbit (*n* = 3) Maximal IOP reduction: ∼5 mmHg observed after 3 h with drug solution. An effect was observed for 5 h. AUC: 11	Youshia et al. (2012)
	1% Tween 80	392 nm, +51 mV	Maximal IOP reduction: ∼6 mmHg observed after 3 h. An effect was observed for 10 h. AUC: 32	
	2% Tween 80	207 nm, +42 mV	Maximal IOP reduction: ∼8 mmHg observed after 3–4 h. An effect was observed for ∼12 h. AUC: 64	
Polymeric nanoparticles; melatonin	Single emulsion followed by solvent evaporation	Drug release	Normotensive rabbit (*n* = 4) Maximal IOP reduction: ∼5 mmHg observed after 2 h with drug solution. An effect was observed for ∼4 h	Musumeci et al. (2013)
	PLGA	130 nm before and 450 nm after freeze drying, 8–9 mV	Maximal IOP reduction: ∼5 mmHg observed after 3 h. An effect was observed for ∼6 h	
	PLGA-PEG	60 nm before and 160 nm after freeze drying, −(23–36) mV	Maximal IOP reduction: ∼5 mmHg observed after 2–3 h. An effect was observed for ∼8 h	
Polymeric nanoparticles; forskolin	Single emulsion followed by solvent evaporation; PLGA coated with chitosan	Drug release, trans-corneal permeation, ocular retention 202 nm, +10 mV	Hypertensive rabbit Lowest IOP value: ∼20 mmHg observed after 1 h with drug suspension. The IOP increased within the next 9 h. Lowest IOP value: ∼16 mmHg observed after 8 h with nanoparticles. The IOP increased within the next 16 h	Khan et al. (2018)
Polymeric nanoparticles; timolol maleate	Desolvation followed by covalent crosslinking; gelatin and glutaraldehyde	Drug release	Hypertensive rabbit (*n* = 4) Lowest IOP value: ∼26 mmHg observed after 12 h with commercial product. AUC (mmHg.h): 332	Shokry et al. (2018)
	crosslinking time: 8 h	782 nm, +20 mV	Lowest IOP value: ∼19 mmHg after 12 h. AUC (mmHg.h): 375	
	crosslinking time: 16 h	206 nm, +13 mV	Lowest IOP value: ∼18 mmHg after 10 h. AUC (mmHg.h): 459	
Polymeric nanoparticles; dorzolamide hydrochloride	Double emulsion followed by solvent evaporation; PLGA with	Drug release, trans-corneal permeation, ocular retention	Normotensive rabbit (*n* = 3) Lowest IOP value: ∼16 mmHg observed after ∼1 h with drug solution. The IOP was lower than control eye for ∼8 h	Warsi et al. (2014)
	PVA	<200 nm	Lowest IOP value: ∼15 mmHg observed after ∼8 h. The IOP was lower than control eye for ∼24 h	
	Vitamin E TPGS	<150 nm	Lowest IOP value: ∼14 mmHg observed after ∼12 h. The IOP was lower than control eye for >24 h	
Polymeric nanoparticles; resveratrol	Ionotropic gelation	Drug release, transcorneal permeation	Normotensive rabbit (n = 3) Lowest IOP value: ∼16–17 mmHg observed after 1.5–2 h with drug dispersion. An effect was observed for ∼4 h	Natesan et al. (2017)
	chitosan, PEG, tripolyphosphate	129 nm	Lowest IOP value: ∼15 mmHg observed after 4 h. An effect was observed for ∼7–8 h	
	additional quercetin	308 nm	Lowest IOP value: ∼14 mmHg observed after 2.5–3 h. An effect was observed for ∼8 h	
Polymeric nanoparticles; carteolol	Ionotropic gelation; chitosan and tripolyphosphate	Drug release, transcorneal permeation, mucin interaction, ocular retention 169 nm	Hypertensive rabbit (*n* = 5) Lowest IOP value: ∼22 mmHg observed after 1 h with drug solution. An effect was observed for 4 h. Lowest IOP value: ∼18 mmHg observed after 2 h with nanoparticles. An effect was observed for ∼12 h	Ameeduzzafar et al. (2014)
Polymeric nanoparticles; latanoprost	Ionotropic gelation; chitosan, hyaluronic acid	314 nm, +30 mV	Normotensive rabbit (*n* = 4) Lowest IOP value: ∼9 mmHg observed after 6 h with drug solution. The IOP gradually increased within the next 6 h. Lowest IOP value: ∼8 mmHg after 6–8 h with nanoparticles. The IOP gradually increased within the next 4 h	Rubenicia et al. (2021)
Liposomes coated with chitosan; timolol maleate	Lipid film-hydration; phosphatidylcholine, cholesterol, and chitosan	Drug release, transcorneal permeation, mucin interaction, ocular retention 151 nm, +16 mV	Normotensive rabbit (*n* = 6). Lowest IOP value: ∼14 mmHg after ∼2 h with commercial product. The IOP gradually increased within the next 4 h. Lowest IOP value: ∼11 mmHg after ∼2 h with nanoparticles. The IOP gradually increased within the next 4 h	Tan et al. (2017)
Liposomes; brinzolamide	Lipid film-hydration; phosphatidylcholine and cholesterol	Drug release, transcorneal permeation 82 nm, −4 mV	Normotensive rabbit (*n* = 6) Lowest IOP value: ∼18–19 mmHg observed after ∼1 h, and lasting for ∼6–8 h, with commercial suspension. The IOP gradually increased within the next 15 h. Lowest IOP value: ∼16 mmHg after ∼1–5 h with nanoparticles. The IOP gradually increased within the next 18 h	Wang et al. (2018)
Polymeric nanoparticles; brimonidine	Single emulsion followed by solvent evaporation	Drug release	Normo- and hypertensive mice (*n* = 5) Lowest IOP value: ∼10–11 mmHg observed after 2 h, with commercial brimonidine tartrate product. The IOP steeply increased within the next 2–4 h. AUC: 26 mmHg	Ibrahim et al. (2015)
	chitosan, poloxamer and lecithin	116 nm, +35 mV	Lowest IOP value: ∼10 mmHg observed after 5–8 h. The IOP gradually increased within the next 8 h. AUC: 80 mmHg	
	alginate, PVA and lecithin	158 nm, −38 mV	Lowest IOP value: ∼10–11 mmHg observed after ∼6 h The IOP gradually increased within the next 10 h. AUC: 72 mmHg	
Liquid crystal nanoparticles; timolol maleate	Single emulsion followed by solvent evaporation; glycerol monoolein and poloxamer	Transcorneal permeation 142 nm, −6 mV	Hypertensive rabbit (*n* = 5) Lowest IOP value: ∼25 mmHg observed after 5 days with commercial product. Lowest IOP value: ∼22 mmHg observed after 6 days, with nanoparticles	Huang et al. (2017)
Dry eye disease				
Polymeric nanoparticles; epigallocatechin gallate	Self-assembly method; gelatin and hyaluronic acid	Drug release, cellular uptake, ocular retention 266 nm, -14 mV	Rabbit with induced DED (*n* = 6) Tear secretion after 3 weeks of treatment: 3–4 mm (drug solution) vs. 6 mm (nanoparticles). The nanoparticles also reduced inflammatory cytokines (TNFα, IL8, IL1β, and IL6) concentration in the cornea and increased the recovery of corneal epithelium thickness	Huang et al. (2018)
Polymeric nanoparticles; dexamethasone	Single emulsion followed by solvent evaporation	Drug release, cellular interaction *in vivo* and *in vitro*	Rabbit with induced DED (*n* = 4–8) The drug suspension was administered 3 times per day, while the nanoparticles were administered every other day. Tear secretion after 2 weeks of treatment: ∼6 mm (drug suspension)	Chen et al. (2021)
	PLGA	Size missing, −7 mV	Tear secretion after 2 weeks of treatment: ∼5 mm	
	additional coating with sebocyte-membrane with integrin-β1	150–200 nm, −13 mV	Tear secretion after 2 weeks of treatment: ∼9 mm This formulation also increased the number of goblet cells, inhibited cell apoptosis, and accelerated epithelial recovery	
Liposomes; cyclosporine A	Lipid film-hydration; cholesterol and phosphatidylcholine or lecithin	Transcorneal permeation, 146–148 nm	Rabbit with induced DED (*n* = 3) Tear secretion after 10 days of treatment: ≤12 mm (commercial emulsion) vs. ≤15 mm (liposomes)	Karn et al. (2014)
Liposomes; tetracycline	Lipid film-hydration; phosphatidylcholine		Rabbit with induced DED (*n* = 3 rabbits/6 eyes) Tear secretion after treatment: ∼17 mm (drug solution) vs. 20 mm (liposomes). The effect of encapsulation was also reflected in the tear break up time used to assess the stability of tear film	Shafaa et al. (2011)
Micelles; cyclosporine A	Desolvation; Cremophor EL, ethanol and glycerol	15–20 nm	Rabbit with induced DED (*n* = 6–8) Tear secretion after treatment: ∼10 mm (commercial emulsion) vs. 13 mm (micelle). The effect of encapsulation was even more pronounced for goblet cell density and conjunctival epithelial morphology	Kang et al. (2016)
Ocular inflammation and endotoxin-induced uveitis				
Polymeric nanoparticles; methylprednisolone acetate	Quasi-emulsion followed by solvent evaporation; Eudragit^®^, PVA, HPMC	Drug release 380 nm	Rabbit with endotoxin-induced uveitis (*n* = 6) After instillation every 6th h, the largest difference in the number of PMN leucocytes in aqueous humor was seen after 36 h: 1175 (drug suspension) vs. 200 (nanoparticles). The effect of encapsulation was also reflected in the protein level and visible signs of inflammation	Adibkia et al. (2007a)
Polymeric nanoparticles; piroxicam	Single emulsion followed by solvent evaporation; Eudragit^®^, PVA, HPMC	Drug release 230–250 nm, +35 mV	Rabbit with endotoxin-induced uveitis (*n* = 6) After instillation every 6th h, the largest difference in the number of PMN leucocytes in aqueous humor was seen after 24 h: 2850 (drug suspension) vs. 1050 (nanoparticles). The effect of encapsulation was also reflected in the visible signs of inflammation	Adibkia et al. (2007b)
Polymeric nanoparticles; aceclofenac	Single emulsion followed by solvent evaporation; Eudragit^®^ and Tween 80	Drug release, trans-corneal permeation 135 nm, +31 mV	Rabbit with inflammation (*n* = 3) The number of PMN leucocytes in aqueous humor 2–3 h after instillation: ∼560–695 (drug solution) vs. ∼360–520 (nanoparticles). The effect of encapsulation was even more pronounced for lid closure scores	Katara and Majumdar, (2013)
Polymeric nanoparticles; aceclofenac	Single emulsion followed by solvent evaporation; Eudragit^®^ and Tween 80	Drug release, trans-corneal permeation 239 nm, +40 mV	Rabbit with inflammation (*n* = 3) The number of PMN leucocytes in aqueous humor 3 h after instillation: ∼740 (drug solution) vs. ∼550 (nanoparticles). The effect of encapsulation was even more pronounced for lid closure scores	Katara et al. (2019)
Polymeric nanoparticles; sodium ibuprofen	Quasi-emulsion followed by solvent evaporation; Eudragit^®^ and Tween 80	51 nm, +35 mV	Rabbit with inflammation (*n* = 4–5) The number of PMN leucocytes in aqueous humor 2 h after instillation: ∼1040 (drug solution) vs. ∼800 (nanoparticles). The effect of encapsulation was also reflected in the protein level and visible signs of inflammation	Bucolo et al. (2002)
Polymeric nanoparticles; celecoxib	Single emulsion followed by desolvation; poly ε-caprolactone and poloxamer	Drug release, trans-corneal permeation 89–191 nm, -(18–32) mV	Rabbit with inflammation (*n* = 3) The number of PMN leucocytes in aqueous humor 4 h after instillation: 633 (drug suspension) vs. 500 (nanoparticles). The effect of encapsulation was also reflected in the protein level and was even more pronounced for lid closure scores	Sharma et al. (2016a)
Solid lipid nanoparticles; celecoxib	Single emulsion followed by solidification; glyceryl monostearate, PVA (lyophilized with mannitol)	Drug release, trans-corneal permeation, ocular retention 199 nm, −16 mV	Rabbit with inflammation (*n* = 3) The number of PMN leucocytes in aqueous humor 4 h after instillation: 617 (drug suspension) vs. 533 (nanoparticles). The effect of encapsulation was also reflected in the protein level and was even more pronounced for lid closure scores	Sharma et al. (2016b)
Micelles; flurbiprofen	Solvent evaporation; 1,2-distearoyl-*sn*-glycero-3-phosphoethanolamine-N-[methoxy (polyethylene glycol)-2000]	Drug release, trans-corneal permeation, cellular interaction, ocular retention 19 nm, −25 mV	Rabbit with inflammation (*n* = 6) The number of PMN leucocytes in aqueous humor 3 h after instillation: ∼18 (commercial flurbiprofen sodium product) vs. ∼12 (micelles). The effect of encapsulation was also reflected in the number of PMN leucocytes in tears and prostaglandin E2 concentration in humor and tears	Weng et al. (2018)
Constriction of the pupil				
Micelles	Self-assembly method		Healthy rabbit (*n* = 6) Duration of pupillary response: 150 min; AUC: 270 mm min, with pilocarpine hydrochloride solution	Pepić et al. (2004)
pilocarpine	Poloxamer	30 nm	Duration of pupillary response: 225 min; AUC: 442 mm min	
pilocarpine hydrochloride	Poloxamer	23 nm	Duration of pupillary response: 180 min; AUC: 297 mm min	
Polymeric nanoparticles; sodium ibuprofen	Quasi-emulsion followed by solvent evaporation; Eudragit^®^, Tween 80	Drug release 48 nm, +18 mV	Healthy rabbit (*n* = 6) Pupil diameter after 1st paracentesis: ∼6.2 mm with commercial ibuprofen lysine product, and ∼6.3 mm with nanoparticles	Pignatello et al. (2002a)
Polymeric nanoparticles; flurbiprofen	Quasi-emulsion followed by solvent evaporation; Eudragit^®^, Tween 80	Drug release 96 nm, +57 mV	Healthy rabbit (*n* = 3) Pupil diameter after paracentesis: ∼6.6 mm with commercial flurbiprofen sodium product, and ∼7.1 mm with nanoparticles	Pignatello et al. (2002b)
Corneal wound healing				
Liquid crystal nanoparticles; pirfenidone	Single emulsion followed by solidification; monolein, oleic acid, poloxamer	Drug release, ocular retention 258 nm, −46 mV	Rabbit with corneal wound (*n* = 5) Nanoparticles accelerated the wound healing process, by ∼ halving the % injured area, and reduced inflammation 82 h after instillation, as reflected in myeloperoxidase activity (∼0.024 vs. ∼ 0.060) and N-acetylglucosaminidase activity (∼0.19 vs. ∼ 0.24) in corneal tissue, when compared to the drug solution	Silva et al. (2019)

AUC, Area under the curve; C_max_, Maximal concentration; DED, Dry Eye Disease; HPMC, Hydroxypropylmethyl cellulose; IL, Interleukin; IOP, Intraocular pressure; PEG, Polyethylene glycol; PLGA, Poly (lactic-co-glycolic) acid; PMN, polymorphonuclear; PVA, Polyvinylalcohol; TNF-α, Tumor necrosis factor-α; Vitamin E TPGS, Tocopheryl polyethylene glycol 1,000 succinate.

Table 4 shows articles on conventional nanoparticles developed for glaucoma, DED, ocular inflammation, endotoxin-induced uveitis, pupillary constriction/dilation, and corneal wound healing. Within each ocular condition, the articles were sorted based on the ranking criteria previously described. Based on 21 articles, glaucoma was the most common condition in the anterior segment of the eye for the development of nanoparticular drug delivery systems. Antiglaucoma drugs entrapped in liposomes (Arroyo et al., 2018), liquid crystal (Li et al., 2013), polymeric (Wadhwa et al., 2010; Bhagav et al., 2011; Khan et al., 2018; Shokry et al., 2018), and solid lipid nanoparticles (Leonardi et al., 2015) showed superior *in vivo* outcomes when compared to the non-encapsulated drugs (Table 3). When timolol maleate was encapsulated into polymeric nanoparticles, the IOP reduction (by ≥ 20%) was prolonged for over 20 h (Shokry et al., 2018). Similarly, encapsulation prolonged the IOP reduction (by ≥ 5 mmHg) for more than 12 h in several other studies (Wadhwa et al., 2010; Bhagav et al., 2011; Leonardi et al., 2015).

Five articles on DED were reviewed. Among these, polymeric gelatin (Huang et al., 2018) and coated PLGA (Chen et al., 2021) nanoparticles enhanced tear production by ∼1.8- and 1.5-fold, respectively. Liposomes (Shafaa et al., 2011; Karn et al., 2014) and micelles (Kang et al., 2016) increased the tear volume by only ∼1.2-fold. The actual tear secretion (in millimeters) after treatment with both encapsulated and non-encapsulated drugs are presented in Table 4. For ocular inflammation and endotoxin-induced uveitis, eight articles were assessed. A noticeable reduction in the number of polymorphonuclear leucocytes in aqueous humor was observed with drug-incorporated nanoparticles compared to the drug in solution or suspension. After a single instillation, the lowest ratio (0.64) was obtained with Eudragit^®^ nanoparticles (Katara and Majumdar, 2013), while the highest ratio (0.86) was obtained with solid lipid nanoparticles (Sharma AK. et al., 2016b). After multiple instillations, Eudragit^®^ nanoparticles resulted in even lower ratios, ranging from 0.17 to 0.60 (Table 4) (Adibkia et al., 2007a; Adibkia et al., 2007b).

Three articles studied pupil constriction or dilation in healthy rabbits using different types of nanoparticles (Table 4). Pepić et al. (2004) showed that micelles only achieved a ∼10% increase in pupillary constriction and a 30 min increase in duration, while Pignatello et al. (2002a) reported that Eudragit^®^ nanoparticles minimally increased the pupil diameter. We found one report of corneal wound healing where liquid crystal nanoparticles were used. The nanoformulation decreased the injured corneal area by ∼50% and reduced inflammation after 82 h of administration compared to non-encapsulated drug (Silva et al., 2019).

In all studied eye conditions, rabbit models were commonly used for investigating the *in vivo* performance of the nanoparticles. The number of animals used per experimental group ranged from 3 to 10 (Table 4). For glaucoma studies, rabbits with both normal as well as elevated IOP were used. For DED, rabbits used were pretreated topically with eye drops containing either 0.1% benzalkonium chloride (Huang et al., 2018; Chen et al., 2021) or 1% atropine sulfate in order to induce disease symptoms (Shafaa et al., 2011; Karn et al., 2014; Kang et al., 2016). Ocular inflammation in rabbits was induced with topical application of arachidonic acid (Bucolo et al., 2002; Katara and Majumdar, 2013; Sharma A. K. et al., 2016a; Sharma AK. et al., 2016b; Weng et al., 2018; Katara et al., 2019) or endotoxin (Adibkia et al., 2007a; Adibkia et al., 2007b). Pupillary constriction was studied in initially healthy animals (Pignatello et al., 2002a; Pignatello et al., 2002b; Pepić et al., 2004) whereas ocular wound was created by topically exposing rabbit eyes to ethanol causing corneal chemical burn (Silva et al., 2019).

As with all research, a risk of bias might be involved when assessing only published studies, as most likely studies with positive therapeutic outcomes are published more often compared to those where no improvements are found. Due to such a bias trend in publication, we might have encountered mostly those studies where nanoparticles showed enhanced therapeutic effect *in vivo* when compared to the non-encapsulated drug.

## 4 Discussion

### 4.1 Nanoencapsulation in Different Ocular Conditions

In the following sections, we present a summary of our main findings. Additionally, characteristics of nanoparticles and encapsulated drug are discussed in relation to their performance in the various conditions of the anterior segment of the eye.

#### 4.1.1 Glaucoma

Glaucoma is an ocular disorder characterized by optic nerve damage in a specific pattern, which if left untreated can potentially result in permanent vision loss (Morrison et al., 2005; Osborne, 2010). Neuroprotection in glaucoma is, thus, a sought-after approach to reduce the disease progression (Vishwaraj et al., 2022). An increase in IOP, usually above 21 mmHg (Heijl et al., 2002), is another critical risk factor of glaucoma. Therefore, effective clinical management of glaucoma can also be achieved by a mean IOP reduction of ∼ ≥ 15% or ≥ 5 mmHg (Akman et al., 2005). Current anti-glaucoma therapies act by either reducing aqueous humor formation or increasing the outflow of the fluid (Aggarwal and Kaur, 2005). Although conventional formulations can reduce the IOP to the normal level (16–18 mmHg), a persistent challenge has been to achieve a prolonged hypotensive effect due to short ocular residence time of the formulations. Pharmacological therapy for glaucoma often implies lifelong treatment. Reducing the frequency of drug administration is crucial to increase patient adherence to therapy and prevent disease progression. The potential of nanoparticles to increase ocular retention of anti-glaucomatous drugs has, therefore, lead to their great interest.

Four studies presented their results as percentage IOP reduction. Among them, the highest improvement of *in vivo* efficacy was obtained upon encapsulation of hydrophilic drugs into liquid crystalline nanoparticles (Li et al., 2013) and conventional liposomes (Arroyo et al., 2018) compared to non-encapsulated drugs. Looking at their physicochemical properties, it seems that small particle size may be beneficial as sizes of ∼15–200 nm (Li et al., 2013; Arroyo et al., 2018) performed better than particle sizes of ∼350 nm (Arroyo et al., 2018). Moreover, surface modification of the nanoparticles seems also to positively influence the therapeutic outcome. For instance, chitosan coated liposomes (Abd-Elsalam and ElKasabgy, 2019) and solid lipid nanoparticles fell into efficacy category 2 (Wang et al., 2014), while plain solid lipid nanoparticles fell into category 3 (Table 3) (Wang et al., 2014).

Seven studies used IOP reduction to report their findings. A distinctive property that appeared from the nanoparticles categorized as “superior” and “good” in terms of efficacy from these studies (category 1 and 2 of Table 3) was positive surface charge. These formulations were either based on the positively charged polymers Eudragit^®^ (Bhagav et al., 2011; Verma et al., 2013) and chitosan (Wadhwa et al., 2010; Manchanda and Sahoo, 2018), or positively charged lipids (Youshia et al., 2012; Leonardi et al., 2015). Similarly, the nanoparticles were small, ranging from ∼118 nm to ∼320 nm (Wadhwa et al., 2010; Bhagav et al., 2011; Youshia et al., 2012; Verma et al., 2013; Leonardi et al., 2015; Manchanda and Sahoo, 2018). However, positive surface charge alone did not guarantee a successful outcome. Positively charged solid lipid nanoparticles have been categorized both as “superior” and “marginal”, depending on the composition of the lipid core (Leonardi et al., 2015). This indicates that other factors than surface charge may also play a role. Especially when palmitic acid was included in the formulation, solid lipid nanoparticles demonstrated “superior” therapeutic outcome (category 1) (Leonardi et al., 2015). Moreover, when positively charged chitosan was modified with hyaluronic acid, the performance was improved; changing from “good” to “superior” (Wadhwa et al., 2010; Manchanda and Sahoo, 2018). According to the authors, this observation could be explained by a synergistic effect for mucoadhesion provided by hyaluronic acid in the formulation (Wadhwa et al., 2010). However, depending on the composition and the overall nanoparticle property, changes in the formulation did not always improve nanoparticle efficacy. Plain PLGA nanoparticles were not more effective than the aqueous drug solution, and modification with polyethylene glycol only marginally improved the therapeutic outcome (Musumeci et al., 2013). When looking at the properties of the encapsulated substance, there were some indications that charged drugs benefitted the most from being encapsulated (Wadhwa et al., 2010; Bhagav et al., 2011; Manchanda and Sahoo, 2018). However, there were some ambiguity as, for example, solid lipid nanoparticles encapsulating melatonin performed “superior” (Leonardi et al., 2015) while incorporation of melatonin into PLGA enhanced efficacy only marginally. The “marginal” performance of PLGA might be explained by its slow melatonin release (Musumeci et al., 2013), as discussed further in section 4.2.1.

Eight studies reported the findings as IOP values in mmHg at different time points (Table 3). In contrast to previous observation (Musumeci et al., 2013), when forskolin (Khan et al., 2018) and dorzolamide hydrochloride were encapsulated into PLGA nanoparticles (Warsi et al., 2014), the performance of the drugs improved substantially (category 1 and 2, respectively). The “superior” PLGA nanoparticles had mucoadhesive chitosan-coating (Khan et al., 2018). The importance of mucoadhesion was also confirmed by Shokry et al. (2018) using gelatin nanoparticles. Smaller gelatin particles (∼206 nm vs. ∼782 nm) performed better *in vivo* (Shokry et al., 2018) following the pattern on particle size. Again, nanoparticles belonging to the “superior” category had positive surface charge (Khan et al., 2018; Shokry et al., 2018). Chitosan-based nanoparticles, however, only performed moderately (category 3) (Ameeduzzafar et al., 2014; Natesan et al., 2017), and after coating with hyaluronic acid, the performance further decreased to “marginal” (Rubenicia et al., 2021). This was surprising as these results contradicted with those from Wadhwa et al. (2010) and Manchanda and Sahoo (2018) discussed earlier. Such inconsistency may be explained by the nature of the encapsulated drugs, where chitosan encapsulation potentially increased the therapeutic effect of a charged/hydrophilic molecule (Wadhwa et al., 2010; Manchanda and Sahoo, 2018) more than an uncharged/lipophilic one (Ameeduzzafar et al., 2014; Natesan et al., 2017; Rubenicia et al., 2021). Liposomes were the least effective delivery system, both plain (Wang et al., 2018) as well as chitosan-coated (Tan et al., 2017), which was contradictory to Arroyo et al. (2018) and Abd-Elsalam and ElKasabgy (2019). Again, drug release could be a possible explanation for such discrepancy further elaborated for Tan et al. (2017) in section 4.2.3.

#### 4.1.2 Dry Eye Disease

DED causes changes in the quality and quantity of the tear film leading to dryness and irritation of the ocular mucosa (Yazdani et al., 2019). Multiple new approaches have been developed in the recent years for effective clinical assessment and treatment of DED (Heidari et al., 2019; Aragona et al., 2021). Here, we have evaluated the therapeutic outcome of nanoparticles in the treatment of DED by using tear secretion as the major parameter.

Compared to non-encapsulated drug, gelatin nanoparticles showed the highest increase in tear production. These nanoparticles were additionally coated with the mucoadhesive polymer hyaluronic acid (Huang et al., 2018). The positive effect of ocular adhesion was also seen in Chen et al. (2021), where both plain and coated PLGA nanoparticles were investigated. Sebocyte membranes engineered to overexpress integrin-β1 that promoted binding to the ocular epithelium was used to coat the PLGA nanoparticles. While coated PLGA nanoparticles increased tear production compared to non-encapsulated drug, plain PLGA nanoparticles decreased the production. However, in this study, dexamethasone suspension as the control was applied more frequently than the PLGA nanoparticles. Therefore, the results might have been underestimated for the latter (Chen et al., 2021). Nanoparticles without obvious adhesive properties, such as liposomes (Shafaa et al., 2011; Karn et al., 2014) and micelles (Kang et al., 2016), only marginally enhanced tear secretion.

#### 4.1.3 Inflammation and Endotoxin-Induced Uveitis

Eye injuries, infection, irritation, and ocular surgery are some of the common causes of ocular inflammation, which can lead to vision-threatening outcomes (Katara and Majumdar, 2013). Ocular inflammation is characterized by infiltration of macrophages and neutrophils of the eye (Katara et al., 2019). Thus, measurement of the number of polymorphonuclear leucocytes in the aqueous humor of the eye can help assess the anti-inflammatory activity of nanoparticles upon their instillation.

Polymeric nanoparticles comprising of Eudragit^®^ most effectively reduced the number of polymorphonuclear leucocytes compared to the non-encapsulated drug (Bucolo et al., 2002; Katara and Majumdar, 2013; Katara et al., 2019). These nanoparticles were positively charged, smaller than 250 nm, and encapsulated both the charged sodium ibuprofen (Bucolo et al., 2002) and uncharged aceclofenac (Katara and Majumdar, 2013; Katara et al., 2019). Poly-ε-caprolactone (Sharma A. K. et al., 2016a) and solid lipid nanoparticles were less effective systems (Sharma AK. et al., 2016b), probably due to a lack of sufficient release of the drug celecoxib from the nanoparticles, as discussed further in section 4.2.1.

In two other studies, Eudragit^®^ nanoparticles were installed multiple times over a course of 36 h (Adibkia et al., 2007a; Adibkia et al., 2007b). Here, encapsulation did not improve the therapeutic outcome of the drugs methylprednisolone acetate and piroxicam for the first 6–12 h, but an increased effect was shown in the 24–36 h time span. Therefore, improved *in vivo* performance of nanoparticles may be revealed after multiple administrations.

#### 4.1.4 Constriction of the Pupil

Surgical or mechanical traumas of the anterior segment of the eye can induce excessive constriction of the pupil (Kulkarni, 1991). The pupil diameter can be increased by pharmacological intervention, which requires a certain availability of the drug at the intraocular level (Pignatello et al., 2002a).

In contrast to earlier promising observations involving Eudragit^®^ nanoparticles (Bucolo et al., 2002; Adibkia et al., 2007a; Adibkia et al., 2007b; Katara and Majumdar, 2013; Katara et al., 2019), Pignatello et al. (2002a) did not manage to further inhibit the pupillary response to surgical trauma with this delivery system. Micelles only achieved a marginal increase of pupillary response reflected in a very low degree of drug encapsulation (Pepić et al., 2004).

#### 4.1.5 Nanoparticle and Drug Characteristics Potentially Influencing the Therapeutic Outcome

Based on our previous discussions, small nanoparticular size and adhesive properties, often related to a positive surface charge, were key factors benefitting the effect of encapsulation. Adhesive properties of nanoparticles could increase their ocular residence time, thereby prolonging the presence of encapsulated drug at the target tissues and, thus, prolonging their action. Larger particle size, on the other hand, may hinder the admittance to the target site (Shokry et al., 2018). The potential benefit of encapsulation might not be the same for charged/hydrophilic drug and an uncharged/lipophilic drug (Pepić et al., 2004). Therefore, it was difficult to independently evaluate the effect of encapsulation when the control and encapsulated drugs were not in the same chemical form (Pignatello et al., 2002b; Ibrahim et al., 2015; Weng et al., 2018). Non-irritant and non-toxic nanoformulations avoid tear stimulation, leading to longer retention times on the corneal surface. Several of the reported studies have shown absence of signs of irritation, inflammation, and histopathological changes in the ocular tissues associated with the nanoformulations. This was shown for different nanoparticular systems as well as excipients; plain (Shafaa et al., 2011; Karn et al., 2014) as well as chitosan-coated (Tan et al., 2017; Abd-Elsalam and ElKasabgy, 2019) liposomes, plain (Ameeduzzafar et al., 2014; Warsi et al., 2014; Huang et al., 2018; Khan et al., 2018) as well as coated (Wadhwa et al., 2010; Ibrahim et al., 2015) polymeric nanoparticles, solid lipid nanoparticles (Wang et al., 2014), nanostructured lipid matrix (Youshia et al., 2012), and liquid crystal nanoparticles (Li et al., 2013; Huang et al., 2017) were well tolerated upon topical instillation.

### 4.2 Associations Between Therapeutic Outcome and *in vitro* Studies

Neither the physicochemical properties of the nanoparticles, such as their size, surface charge, type of core and surface coating, nor the type of encapsulated drug, could solely determine nanoparticles’ behavior *in vivo*. For instance, liposomes showed both “superior” (Arroyo et al., 2018) and “marginal” therapeutic efficacy in glaucomatous rabbits. Similarly, PLGA formulations were the best (Warsi et al., 2014; Khan et al., 2018) as well as poor (Musumeci et al., 2013) performing systems *in vivo*. Eudragit^®^ nanoparticles displayed “good” therapeutic efficacy in rabbits with glaucoma (Bhagav et al., 2011; Verma et al., 2013) and inflammation (Bucolo et al., 2002; Katara and Majumdar, 2013; Katara et al., 2019), but the *in vivo* effect was poor in pupillary constriction/dilation (Pignatello et al., 2002a). We, therefore, searched for a more complex interplay between the properties of nanoparticles and encapsulated drug, and their therapeutic efficacy, which might be revealed in their drug release, mucoadhesion or transcorneal permeation properties, often measured *in vitro/ex vivo* as part of the development work.

#### 4.2.1 Drug Release

To achieve prolonged therapeutic effect, as desired for ocular drug delivery, the drug should be released from the nanoparticles in a sustained manner. Several studies have shown correlation of improved therapeutic outcome of nanoencapsulated drug with sustained drug release *in vitro* (Li et al., 2013; Ameeduzzafar et al., 2014; Tan et al., 2017; Khan et al., 2018; Wang et al., 2018). Such correlation was also demonstrated among different nanoparticular compositions, made with varying proportions of secondary emulsifier and polymer. The Eudragit^®^ formulation with higher burst release followed by a fast drug release *in vitro* had a shorter duration of drug action compared to the one that displayed a sustained release profile (IOP reduction for ∼36 h vs. 72 h) (Bhagav et al., 2011). Nevertheless, converse correlation has also been demonstrated. Solid lipid nanoparticles based on heterolipids, also termed nanostructured lipid matrices (Youshia et al., 2012), and chitosan nanoparticles (Manchanda and Sahoo, 2018) with fast drug release displayed a more pronounced and longer lasting IOP reduction. The reason for this discrepancy might be that drug release first and foremost must align with the residence time of the respective nanoparticle. In other words, time and place for drug release should coincide for an optimal therapeutic effect. A consequence of non-alignment was encountered in Abd-Elsalam and ElKasabgy (2019). Here, an *in vivo* therapeutic effect was achieved for 8 h indicating an even shorter residence time. However, the slowest releasing nanoparticle only released ∼50% of the drug *in vitro* within these 8 h. An increased pharmacological effect was observed when the drug release was increased to ∼70%. The therapeutic effect of this formulation could have increased had a higher amount of drug been released within the ocular residence time. In another study, there was only marginal improvement in IOP reduction with polyethylene glycol modified PLGA nanoparticles compared to the aqueous melatonin solution. Such poor performance was evident in the non-alignment of drug release from nanoparticles *in vitro* and the time of effect *in vivo*. The results showed that only 20% of the drug was released after 24 h from the nanoparticles (Musumeci et al., 2013). A similar situation was encountered in Sharma A. K. et al. (2016a) and Sharma AK. et al. (2016b) where the number of polymorphonuclear leucocytes were measured after 4 h, however, only ∼20% of the drug was released by that time.

Drug release is an important factor for influencing therapeutic outcome. This was also demonstrated in Warsi et al. (2014), where various PLGA nanoparticles had similar drug release rates that were reflected by their therapeutic effects. However, the case is not always that simple. Gelatin nanoparticles crosslinked for 16 or 8 h had almost superimposable release profiles, despite different therapeutic outcomes (Shokry et al., 2018) attributed to the positive effect of small particle size (∼206 nm vs. ∼782 nm). And in Leonardi et al. (2015), the release of melatonin from various solid lipid nanoparticles at different time points could not be used to explain the *in vivo* outcome. The findings from Shokry et al. (2018) and Leonardi et al. (2015) indicated that additional parameters besides drug release might also be important, such as residence time and transcorneal drug permeability.

#### 4.2.2 Ocular Surface Residence Time

Increased ocular surface residence time of nanoparticles will counteract the highly efficient ocular lacrimal drainage system. As an estimation of the *in vivo* ocular residence time, the interaction between mucus and the nanoparticles has been tested *in vitro* (Wadhwa et al., 2010; Ameeduzzafar et al., 2014; Tan et al., 2017; Manchanda and Sahoo, 2018; Abd-Elsalam and ElKasabgy, 2019). The mucin-particle method is commonly used, where changes in zeta potential and the mean particle size of nanoparticles are measured following mixing with negatively charged mucin (de Sá et al., 2015; Tan et al., 2017; Abd-Elsalam and ElKasabgy, 2019).

Manchanda and Sahoo (2018) examined the mucus-interaction of various chitosan nanoparticles and found associations with their *in vivo* therapeutic performance. When chitosan nanoparticles were coated with hyaluronic acid, the therapeutic effect increased (Wadhwa et al., 2010). The modification did not affect the *in vitro* drug release, which remained almost unchanged. The mucus-interaction increased slightly, although this increase (91% vs. 87%) was not significant enough to truly reflect the pronounced therapeutic benefit of this modification. This shows that *in vitro* mucus interaction assays alone do not sufficiently predict the *in vivo* ocular retention and, thus, the therapeutic effect of the nanoparticles. Some studies have used gamma scintigraphy for better prediction of nanoparticle behavior (Ameeduzzafar et al., 2014; Warsi et al., 2014; Tan et al., 2017; Khan et al., 2018). This technique, if available, gives sufficient information to design an effective delivery system where the drug release rate is targeted at the actual retention time of the nanoparticle. In the above-mentioned studies, however, *in vivo* retention was only traced for 10 min (Tan et al., 2017), 30 min (Ameeduzzafar et al., 2014; Warsi et al., 2014) or up to 6 h (Khan et al., 2018). Although the latter is a long time, it did not match the drug release, which was monitored for at least 12 h.

#### 4.2.3 Transcorneal Drug Permeability

Nanoencapsulation can also facilitate co-delivery of drug and permeation enhancers. The multifunctional polymer chitosan has permeation enhancing effect in addition to being mucoadhesive (Wadhwa et al., 2010; Ameeduzzafar et al., 2014; Tan et al., 2017; Khan et al., 2018). Other examples include surfactants, such as glyceryl monoolein (Li et al., 2013) or vitamin E tocopheryl polyethylene glycol 1,000 succinate (TPGS) (Warsi et al., 2014). Indeed, a correlation between the therapeutic outcome of nanoparticles and permeation through excised goat or rabbit cornea has been demonstrated in several studies. The *ex vivo* transcorneal transport of drug from nanoparticles was higher than from drug solution or suspension (Li et al., 2013; Ameeduzzafar et al., 2014; Tan et al., 2017; Khan et al., 2018).

On the contrary, the *ex vivo* enhanced permeability of vitamin E TPGS over polyvinyl alcohol-incorporated PLGA nanoparticles failed to corroborate *in vivo* results (Warsi et al., 2014)*.* Their performance *in vivo* was comparable as were their drug release rates as discussed earlier. This may indicate an overestimation of permeation in *ex vivo* experiments. Similar observations were made in Manchanda and Sahoo (2018), where the difference in *ex vivo* transcorneal transport after 2 h was much larger than the difference in therapeutic outcome, which was better reflected in *in vitro* drug release and mucus-interaction. Thus, the *ex vivo* permeation assay is probably limited to comparing and ranking various nanoparticles and has less merit for predicting their *in vivo* performance. Advantages and limitations of *in vitro* and *ex vivo* corneal permeation assays have been reviewed previously (Agarwal and Rupenthal, 2016). A lack of alignment between drug release and permeation was apparent in Tan et al. (2017). There, close to 100% of the drug permeated within 6 h, while only ∼60% was released in the same period in the *in vitro* drug release experiment.

There were also examples where the relationship between *ex vivo* permeation and therapeutic outcome was ambiguous. In one instance, two different types of nanoparticles (chitosan and hyaluronic acid modified chitosan) with similar drug release rates were investigated. An improved therapeutic effect was observed with hyaluronic acid modified chitosan nanoparticles. The mucus-interaction predicted to some degree this improvement. The permeability assay was also capable of predicting this improvement, but only after 4 h and not for the first 2 h of the *ex vivo* study (Wadhwa et al., 2010). Additionally, the benefit of nanoencapsulation was overestimated at 4 h. In another study, permeation was lower for the encapsulated drug although the therapeutic outcome improved (Wang et al., 2014).

### 4.3 Challenges With Clinical Translation

Our investigation revealed that the *in vivo* correlation of commonly used *in vitro* tests during development and characterization of nanoparticles was limited. At present, these methodologies are limited in their usefulness unless accompanied by an *in vivo* proof of concept. Although each individual test can be used for comparative evaluation between different types of nanoparticles, we observed that the test results of various *in vitro* and *ex vivo* studies could not always be successfully combined. Therefore, designing a nanoparticle whose *in vivo* drug release aligns with the residence time *in vivo* has posed a challenge. Even after optimization of the formulation, the clinical translation of nanoparticles is faced with several technological, development and production issues (Weng et al., 2017; Jumelle et al., 2020). In addition, not every nanoparticle with promising therapeutic outcome in animal models performs well in clinical trials. One problem is that there are anatomical and physiological differences between humans and commonly used animal models, such as mice, rats and rabbits (Morrison et al., 2005). For instance, rabbit ocular anatomy, although being similar and comparable to human, does not completely mimic the latter. Rabbit eyes have higher mucus production, higher surface sensitivity and lower rate of blinking, which can result in better drug retention and drug penetration compared to human eyes, thus, resulting in overestimation of therapeutic outcome that cannot be extrapolated to humans (Destruel et al., 2017; Weng et al., 2017).

## 5 Conclusion

In the present study, we have evaluated and ranked various types of nanoparticles based on their therapeutic merits compared to non-encapsulated drug for ocular delivery. The majority of the studies demonstrated some improved efficacy of drugs after encapsulation, however, to variable degrees. The greatest achievement was quite substantial. For instance, the successful nanoparticles prolonged IOP reduction for over 20 h. Other promising nanoformulations increased tear production by ∼80%. Similarly, nanoparticles reduced polymorphonuclear leucocytes by ∼36% on single administration, which increased to an impressive ∼83% after multiple instillations. We have noticed trends that small and mucoadhesive nanoparticles, often caused by a positive surface charge, might be beneficial. However, this picture is ambiguous, possibly due to the complex interplay between the physicochemical properties of the drug along with the core and surface properties of the nanoparticles. This interplay was sometimes revealed in *in vitro* drug release, *in vitro* mucus interaction and/or *ex vivo* permeation tests.

Future work should be directed towards designing nanoparticles by systematic evaluation of one formulation parameter at a time. It is also crucial that the drug release aligns with formulation residence time *in vivo*. Additionally, development of more *in vivo* relevant *in vitro* assays, particularly for adhesion and permeability, may improve the characterization of nanoparticles for a particular drug and for a specific condition. Rational extrapolation of data from relevant *in vitro* experiments would then be used to predict *in vivo* behavior, thereby minimizing the need for animal testing, which is already limited due to ethical, economic, and technical reasons.

## Data Availability

The original contributions presented in the study are included in the article/Supplementary Material, further inquiries can be directed to the corresponding author.
